# Three new species and one new record of the genus *Siphunculina* from China (Diptera, Chloropidae)

**DOI:** 10.3897/zookeys.687.13156

**Published:** 2017-08-02

**Authors:** Xiao-Yan Liu, Emilia P. Nartshuk, Ding Yang

**Affiliations:** 1 Hubei Insect Resources Utilization and Sustainable Pest Management Key Laboratory, College of Plant Science and Technology, Huazhong Agricultural University, Wuhan 430070, China; 2 Zoological Institute, Russian Academy of Sciences, St. Petersburg 199034, Russia; 3 Department of Entomology, China Agricultural University, Beijing 100193, China

**Keywords:** China, Chloropidae, Diptera, new species, *Siphunculina*, Taxonomy

## Abstract

Three new species of the genus *Siphunculina* Rondani from China, *S. bulbifera* **sp. n.**, *S. scalpriformis* **sp. n.**, and *S. shangyongensis* **sp. n.**, are described and illustrated. One species, *S. funicola* (de Meijere), is reported from China for the first time. A key to the species of genus *Siphunculina* from China is given.

## Introduction

The genus *Siphunculina* was erected by Rondani (1856). It belongs to the *Aphanotrigonum* genus group of the subfamily Oscinellinae (Andersson 1977). There are 34 species known from the world, of which 17 species are distributed in the Oriental Region, ten species in the Palaearctic Region, eleven species in the Afrotropical, three species in the Australian, only one species is known to occur in the Nearctic and Neotropical Regions (Cherian 1970, 1977; Sabrosky 1977, 1980, 1989; Kanmiya 1982, 1989, 1994; Nartshuk 1984, 2005, 2007; Ismay and Nartshuk 2000; Merz 2008; Iwasa et al. 2013). Adults of some species are attracted to decaying meat, wounds, scratches, mucous membranes, eyes, lips, moist skin, in-between toes, sweat, and other secretions of the body and are suspected of mechanically transmitting pathogenic organisms to man and domestic animals (Graham-Smith 1930; Chansang et al. 2010). The larvae can be found in birds’ nests, excrement, or dead animals, which are saprophilous or scatophagous (Kanmiya 1983; Ferrar 1987; Ismay and Nartshuk 2000).

To date, five species are known to occur in China, of which four are known from Taiwan and two species are distributed in mainland China. In this paper, three new species of the genus *Siphunculina* from China, *S. bulbifera* sp. n., *S. scalpriformis* sp. n. and *S. shangyongensis* sp. n., are described and illustrated. One species, *S. funicola* (de Meijere), is newly recorded from China. A key to the species of genus *Siphunculina* from China is given.

## Materials and methods

Specimens were studied and illustrated with ZEISS Stemi 2000–c. Genitalic preparations were made by macerating the apical portion of abdomen in warm 10% NaOH for 17–20 min, after examination it was transferred to fresh glycerine and stored in a microvial pinned below the specimen. Specimens are deposited in the Entomological Museum of China Agricultural University (CAU), Beijing. The morphological nomenclature follows Cumming and Wood (2009). The following abbreviations are used:

**bas** basiphallus;

**cerc** cercus;

**dis** distiphallus;

**dm-cu** discal medial-cubital crossvein;

**ep** epandrium;

**gon** gonite;

**hyp** hypandrium;

**phal** phallapodeme;

**r-m** radio-medial crossvein;

**sur** surstylus.

## Taxonomy

### Family Chloropidae

#### Genus Siphunculina

Taxon classificationAnimaliaDipteraChloropidae

Rondani, 1856

Siphunculina Rondani, 1856: 128. Type species: Siphunculina brevinervis Rondani, 1856 (= Siphonella aenea Macquart, 1835), by original designation.
Microneurum Becker, 1903: 152. Type species: Microneurum maculifrons Becker, 1903 (= Oscinis ornatifrons Loew, 1858), by monotypy.
Liomicroneurum Enderlein, 1911: 230. Type species: Siphonella funicola de Meijere, 1905, by original designation.

##### Diagnosis.

Head with vibrissal angle more or less distinctly produced beyond eye; face with deeply concave antennal foveae and a distinct median carina reaching epistoma; cephalic setae and setulae generally short; wing with veins R_1_ and R_2+3_ extremely closed on basal portion, vein R_2+3_ very short, length of 2nd costal sector extremely shorter than the 3rd sector; femoral and tibial organs absent (Kanmiya 1983, 1994).

##### Distribution.

Widespread world-wide distribution, see Nartshuk (2012). China: Beijing, Zhejiang, Hainan, Guizhou, Yunnan, Taiwan.

##### Key to species of *Siphunculina* from China

**Table:** 

1	Cephalic setae and setulae black or brown	**2**
–	Cephalic setae and setulae light yellow or yellow	**7**
2	Ocellar triangle shiny except for ocellar tubercle, without microtomentum; frons, ocellar triangle and scutum marked out by reticulate patterns with alternating microtomentose and bare maculae	***S. striolata* (Wiedemann)**
–	Ocellar triangle not entirely shiny, partly or entirely microtomentose; frons, ocellar triangle and scutum not marked out by alternating microtomentose and bare maculae	**3**
3	Notopleurals 1+2; apical scutellar seta as long as scutellum (Fig. 20)	***S. funicola* (de Meijere)**
–	Notopleurals 1+1; apical scutellar seta shorter than scutellum	**4**
4	Two pairs of scutellar setae; 3rd costal sector 2 times as long as 2nd sector (Fig. 2)	***S. bulbifera* sp. n.**
–	Three pairs of scutellar setae; 3rd costal sector 3-4 times as long as 2nd sector	**5**
5	Ocellar triangle nearly or completely reaching anterior margin of frons, with a median groove	***S. nitidissima* Kanmiya**
–	Ocellar triangle ending slightly but distinctly before anterior margin of frons, without median groove	**6**
6	Hind tibia yellow with largely infuscate maculae medially; tarsi entirely yellow; surstylus as long as the epandrium in lateral view	***S. bella* Kanmiya**
–	Hind tibia yellow except for middle 1/3 brown; tarsi yellow except for hind tarsomeres 2-4 brown; surstylus 0.6 times as long as the epandrium in lateral view (Figs 13, 16)	***S. shangyongensis* sp. n.**
7	Ocellar triangle extending to middle or slightly over middle of frons	**8**
–	Ocellar triangle extending near or completely to anterior margin of frons	***S. minima* (de Meijere)**
8	Ocellar triangle microtomentose except for area in front of ocelli and on both sides of ocellar tubercle shiny (Fig. 8)	***S. scalpriformis* sp. n.**
–	Ocellar triangle shiny with 2 microtomentose patches extending from nearly mediolateral of triangle to anterior ocellus	***S. fasciata* Cherian**

#### Siphunculina bulbifera sp. n.

Taxon classificationAnimaliaDipteraChloropidae

http://zoobank.org/2A36E011-58B7-47A8-9B00-BEABB0027FFE

Figs 1–2 , 3–6

##### Diagnosis.

Ocellar triangle smooth, shiny, reaching anterior margin of frons, with pointed apex. Scutellum with two pairs of scutellar setae on small tubercles. Cephalic and thoracic setae and setulae black. Notopleurals 1+1. Distal 2/3 of gonite long globose.

**Figures 1–2.**
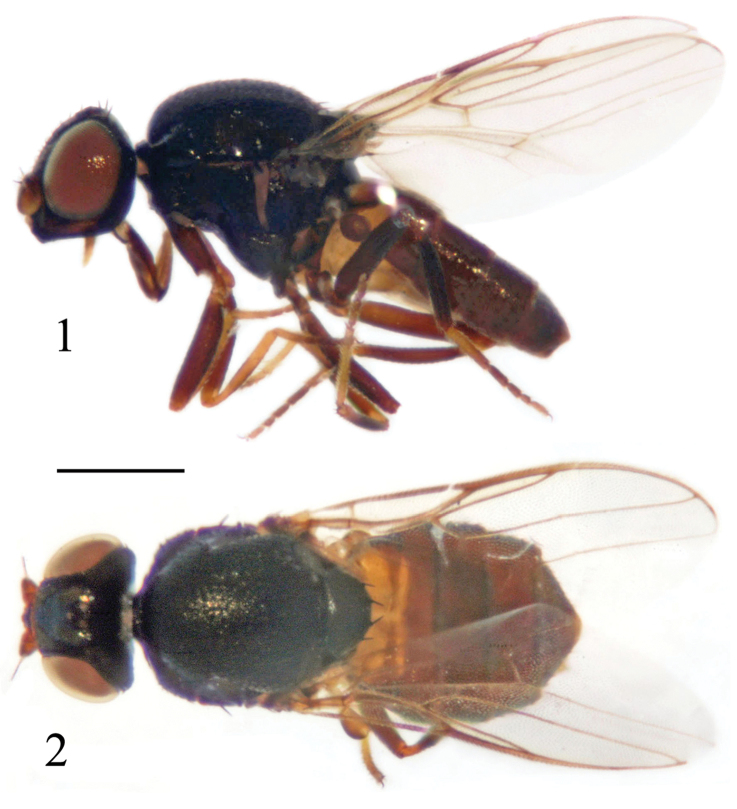
*Siphunculina bulbifera* sp. n., holotype. habitus, **1** lateral view **2** dorsal view. Scale bar 0.05 mm.

##### Description.

***Holotype. Male***. Body length 2.2 mm, wing length 1.6 mm.

*Head* black, 0.8 times as long as high in profile, as wide as thorax; face somewhat concave in lateral view, facial carina distinct, broad; frons as long as wide, projecting slightly in front of eye; gena broad, 0.5 times as wide as first flagellomere; parafacial linear; vibrissal angle distinctly produced beyond eye by subequal length to gena-width. Ocellar triangle smooth, shiny, reaching anterior margin of frons, with pointed apex; ocellar tubercle black. Cephalic setae and setulae black. Antenna yellow except for distodorsal 1/2 of first flagellomere brown, with thick grayish microtomentum; first flagellomere 0.6 times as long as wide; arista black except for basal segment yellow, with short pubescence. Proboscis brown to yellow with yellow setulae; palpus brown to yellow with yellow setulae.

*Thorax* black with gray microtomentum, evenly covered with short setulae. Scutum as long as wide. Thoracic pleuron shiny black. Scutellum 0.55 times as long as wide; two pairs of scutellar setae on small tubercles; apical scutellar seta short, 0.3 times as long as scutellum. Setae and setulae on thorax black; notopleurals 1+1, developed. Legs brown except for basal portion and distal 1/3 of fore tibia, both ends of mid and hind tibiae, fore and hind tarsomeres 1, mid tarsomeres 1-2 yellow. Setulae on legs brown. Wing 2.3 times as long as wide, hyaline; veins yellowish brown. Relative lengths of 2nd : 3rd : 4th costal sections = 5 : 10 : 3; cross-veins r-m and dm-cu not approximated, r-m at basal 0.65 of discal medial cell. Halter brown.

*Abdomen* shiny brown except for tergite 1 yellow with brown distally; venter yellow. Setulae on abdomen black.

**Figures 3–6.**
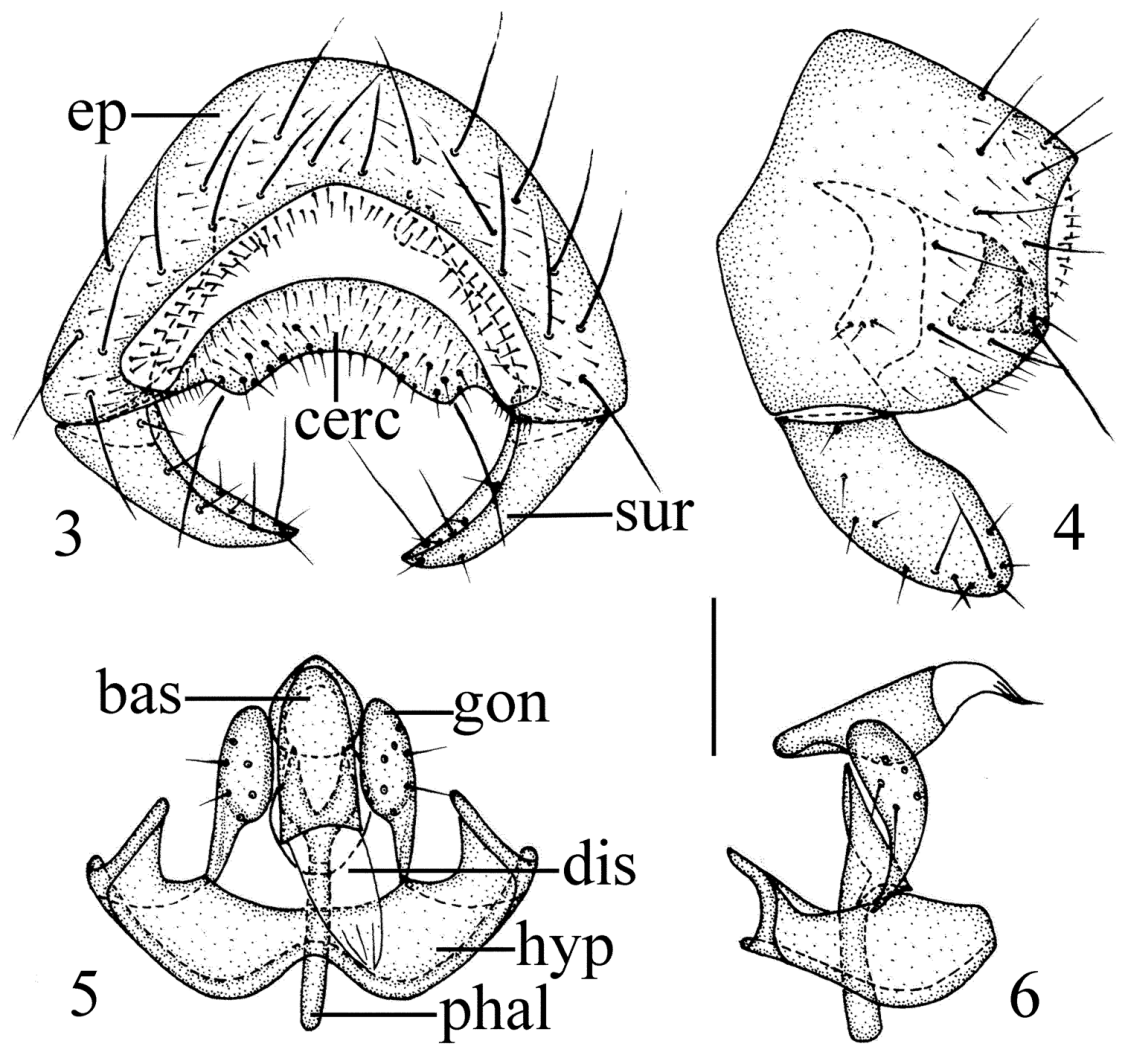
*Siphunculina bulbifera* sp. n., holotype. **3** epandrium, posterior view **4** epandrium, lateral view **5** hypandrium and phallic complex, ventral view **6** hypandrium and phallic complex, lateral view. Scale bar: 0.1 mm.

*Male genitalia* (Figs 3–6): Epandrium blackish brown with long brown setulae; surstylus distinctly shorter than epandrium in lateral view. Cercus short and broad, with moderately concave ventral margin in dorsal view. Pre- and postgonites fused, basal 1/3 narrow, distal 2/3 long globose; basiphallus longer than wide, cylindrical; distiphallus short, membranous; phallapodeme long, with basal stalk narrow in lateral view. Hypandrium broad, with two basal rounded projections, arms free and short, apex with internal projection long, external projection short.

***Female*.** Unknown.

##### Type material.

Holotype, ♂, China: Beijing: Shidu, 1-2. VI. 2009, leg. Dan Zhou (photographed and genitalia prepared). Paratype, 2 ♂♂, same locality as holotype, 1-2. VII. 2009, leg. Jinjing Wang (in 75% alcohol, deposited in CAU).

##### Distribution.

China (Beijing).

##### Remarks.

The new species is somewhat similar to *S. nitidissima* Kanmiya, but can be separated from the latter by the following features: ocellar triangle smooth without median groove, two pairs of scutellar setae on small tubercles, and gonite has narrow base, its distal 2/3 long and globose; in *S. nitidissima*, the ocellar triangle has a median groove, the scutellum has three pairs of scutellar setae on small tubercles, and the gonite is finger-like (Kanmiya 1982).

##### Etymology.

The specific name is from the Latin *bulbifera* (“bulbiform”), referring to the shape of gonite.

#### Siphunculina scalpriformis sp. n.

Taxon classificationAnimaliaDipteraChloropidae

http://zoobank.org/3DFBB88D-1403-4FD0-92D6-02AEC4090064

Figs 7–8 , 9–12

##### Diagnosis.

Ocellar triangle black with gray microtomentum except for area in front of ocelli and on both sides of ocellar tubercle shiny, reaching anterior 0.6 of frons. Scutellum with three pairs of scutellar setae. Cephalic and thoracic setae and setulae yellow. Notopleurals 1+1. Gonite knife-like, incised on basal 1/3 of each inner margin.

**Figures 7–8.**
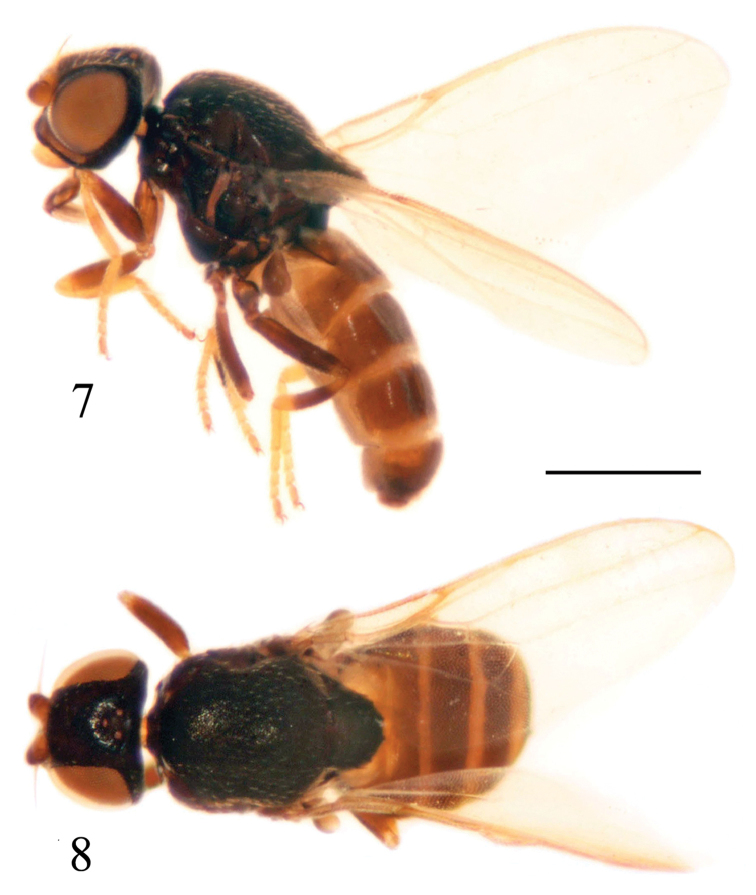
*Siphunculina scalpriformis* sp. n., holotype. habitus **7** lateral view **8** dorsal view. Scale bar 0.05 mm.

##### Description.

***Holotype. Male***. Body length 1.9–2.0 mm, wing length 1.5–1.6 mm.

*Head* black with gray microtomentum, 0.7 times as long as high in profile, as wide as thorax; face somewhat concave in lateral view, facial carina narrow; frons 0.9 times as long as wide, projecting slightly in front of eye; gena yellow except for ventral 1/3 black, broad, 0.5 times as wide as first flagellomere; parafacial linear; vibrissal angle weakly produced beyond eye. Ocellar triangle partly microtomentose, area in front of ocelli and on both sides of ocellar tubercle shiny, its apex reaching anterior 0.6 of frons, with slightly pointed apex; ocellar tubercle black. Cephalic setae and setulae yellow. Antenna yellow except for dorsal margin of first flagellomere brown, with thick grayish microtomentum; first flagellomere 0.75 times as long as wide; arista black except for basal segment brownish, with short pubescence. Proboscis black to yellowish brown with yellow setulae; palpus yellow with yellow setulae.

*Thorax* black with gray microtomentum, evenly covered with short setulae. Scutum as long as wide. Thoracic pleuron shiny black without microtomentum. Scutellum 0.6 times as long as wide; 3 pairs of scutellar setae on small tubercles; apical scutellar seta short, 0.5 times as long as scutellum. Setae and setulae on thorax yellow; notopleurals 1+1, developed. Legs with coxae black, femora black with distal tips yellow, fore tibia yellow with weakly infuscated medially, mid and hind tibiae black with both tips yellow, tarsi yellow. Setulae on legs yellow. Wing 2.2 times as long as wide, hyaline; veins brownish. Relative lengths of 2nd : 3rd : 4th costal sections = 6 : 17 : 4; cross-veins r-m and dm-cu not approximated, r-m at basal 0.65 of discal medial cell. Halter brown.

*Abdomen* shiny brown; venter yellow. Setulae on abdomen black.

**Figures 9–12.**
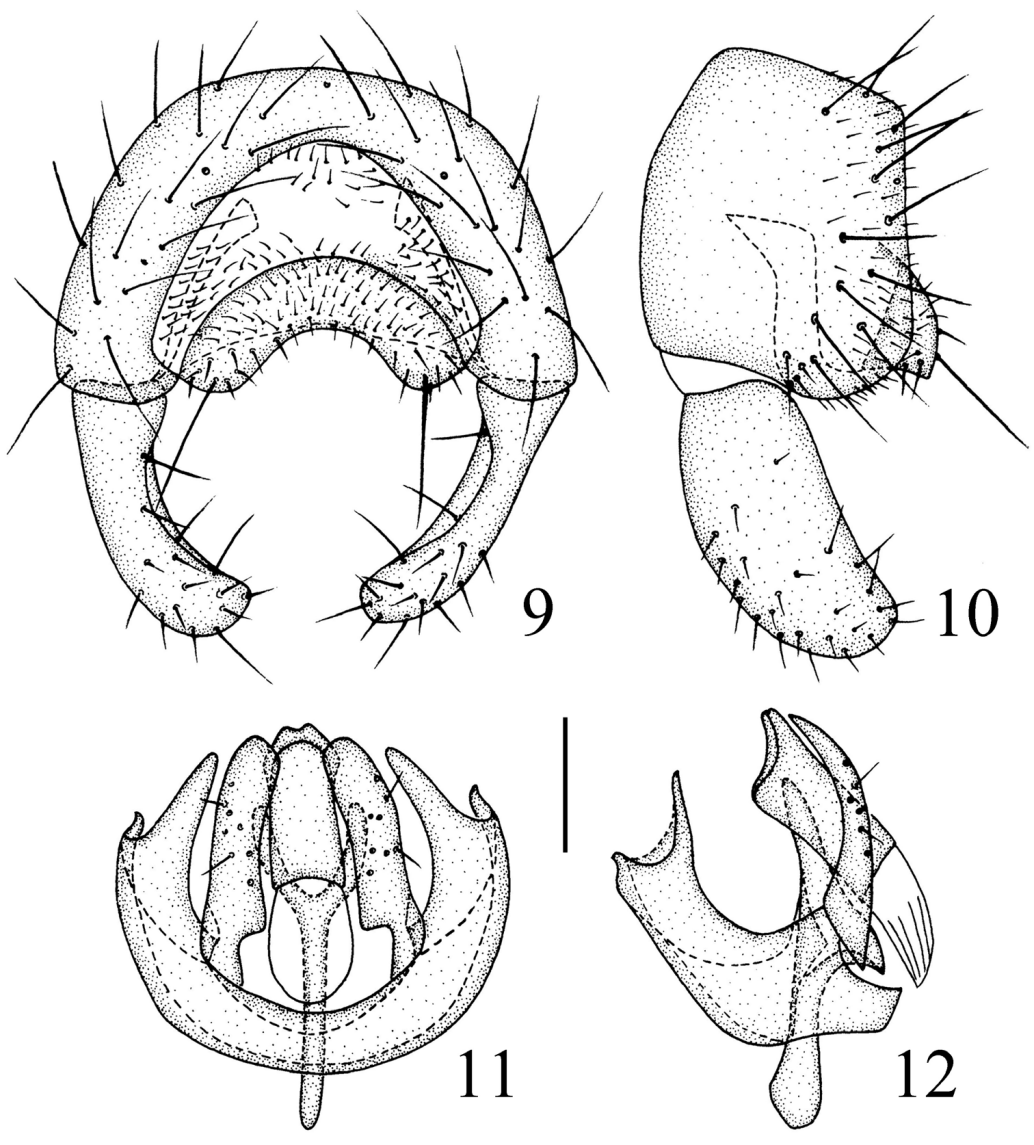
*Siphunculina scalpriformis* sp. n., holotype. **9** epandrium, posterior view **10** epandrium, lateral view **11** hypandrium and phallic complex, ventral view **12** hypandrium and phallic complex, lateral view. Scale bar: 0.1 mm.

*Male genitalia* (Figs 9–12): Epandrium blackish brown with long brown setulae; surstylus as long as epandrium in lateral view. Cercus short and broad, with moderately concave ventral margin in dorsal view. Pre- and postgonites fused, knife-like, incised on basal 1/3 of each inner margin; basiphallus longer than wide, cylindrical; distiphallus short, membranous; phallapodeme long, with basal stalk narrow in lateral view. Hypandrium broad, without two basal rounded projections, arms free and long, apex with internal projection long, external projection short.

***Female*.** Unknown.

##### Type material.

Holotype, ♂, China: Guizhou: Maolan, Yaoqu, 30. V. 2010, leg. Dan Zhou (photographed and genitalia prepared). Paratype, 2 ♂♂, same data as holotype (in 75% alcohol, deposited in CAU).

##### Distribution.

China (Guizhou).

##### Remarks.

The new species is somewhat similar to *S. minima* (de Meijere), but can be separated from the latter by the following features: ocellar triangle reaching anterior 0.6 of frons, gena 0.5 times as wide as first flagellomere; in *S. minima*, the ocellar triangle reaches anterior 0.9 of frons, the gena is as wide as the first flagellomere (Kanmiya 1982).

##### Etymology.

The specific name is from the Latin scalpriformis (“knife-like”), referring to the shape of gonite.

#### Siphunculina shangyongensis sp. n.

Taxon classificationAnimaliaDipteraChloropidae

http://zoobank.org/67F6EFAD-6BE8-4820-B886-7A39745E3727

Figs 13–14 , 15–18

##### Diagnosis.

Ocellar triangle black, smooth, shiny, reaching anterior 0.9 of frons, with slightly pointed apex. Scutellum with three pairs of scutellar setae on small tubercles. Cephalic and thoracic setae and setulae black. Notopleurals 1+1. Gonite nearly rectangular, slightly narrowed basally.

**Figures 13–14.**
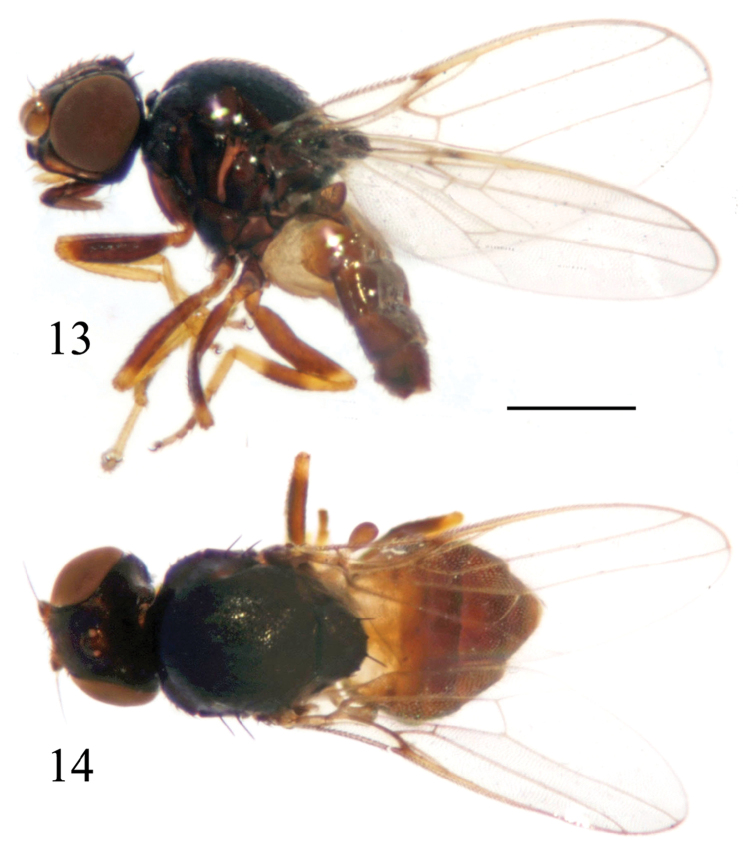
*Siphunculina shangyongensis* sp. n., holotype. habitus, **13** lateral view **14** dorsal view. Scale bar: 0.05 mm.

##### Description.

***Holotype. Male***. Body length 1.8 mm, wing length 1.6 mm.

*Head* black with gray microtomentum, 0.75 times as long as high in profile, as wide as thorax; face somewhat concave in lateral view, facial carina distinct; frons brown, 0.9 times as long as wide, projecting only slightly in front of eye; gena broad, 0.5 times as wide as first flagellomere, yellowish brown except for ventral margin black; parafacial indistinct; vibrissal angle distinctly produced beyond eye by subequal length to gena-width. Ocellar triangle black, smooth, shiny, reaching anterior 0.9 of frons, with slightly pointed apex; ocellar tubercle brown. Cephalic setae and setulae black. Antenna yellow with thick grayish microtomentum except for distodorsal margin of first flagellomere black; first flagellomere as long as wide; arista black except for basal segment yellow, with short brown pubescence. Proboscis brown with brown setulae; palpus yellow with brown setulae.

*Thorax* black, evenly covered with short setulae. Scutum 1.1 times as long as wide. Thoracic pleuron shiny brown without microtomentum. Scutellum black, 0.6 times as long as wide; 3 pairs of scutellar setae on small tubercles; apical scutellar seta 0.5 times as long as scutellum. Setae and setulae on thorax black; notopleurals 1+1, developed. Legs with coxae brown, femora brown with distal tips yellow, tibiae yellow with middle 1/3 of mid tibia brownish, middle 1/3 of hind tibia brown, tarsi yellow with hind tarsomeres 2-4 brown. Setulae on legs brown. Wing 2.2 times as long as wide, hyaline; veins yellowish brown. Relative lengths of 2nd : 3rd : 4th costal sections = 3 : 11 : 3; cross-veins r-m and dm-cu not approximated, r-m at basal 0.6 of discal medial cell. Halter brown.

*Abdomen* shiny brown except for tergite 1 yellow; venter yellow. Setulae on abdomen black.

**Figures 15–18.**
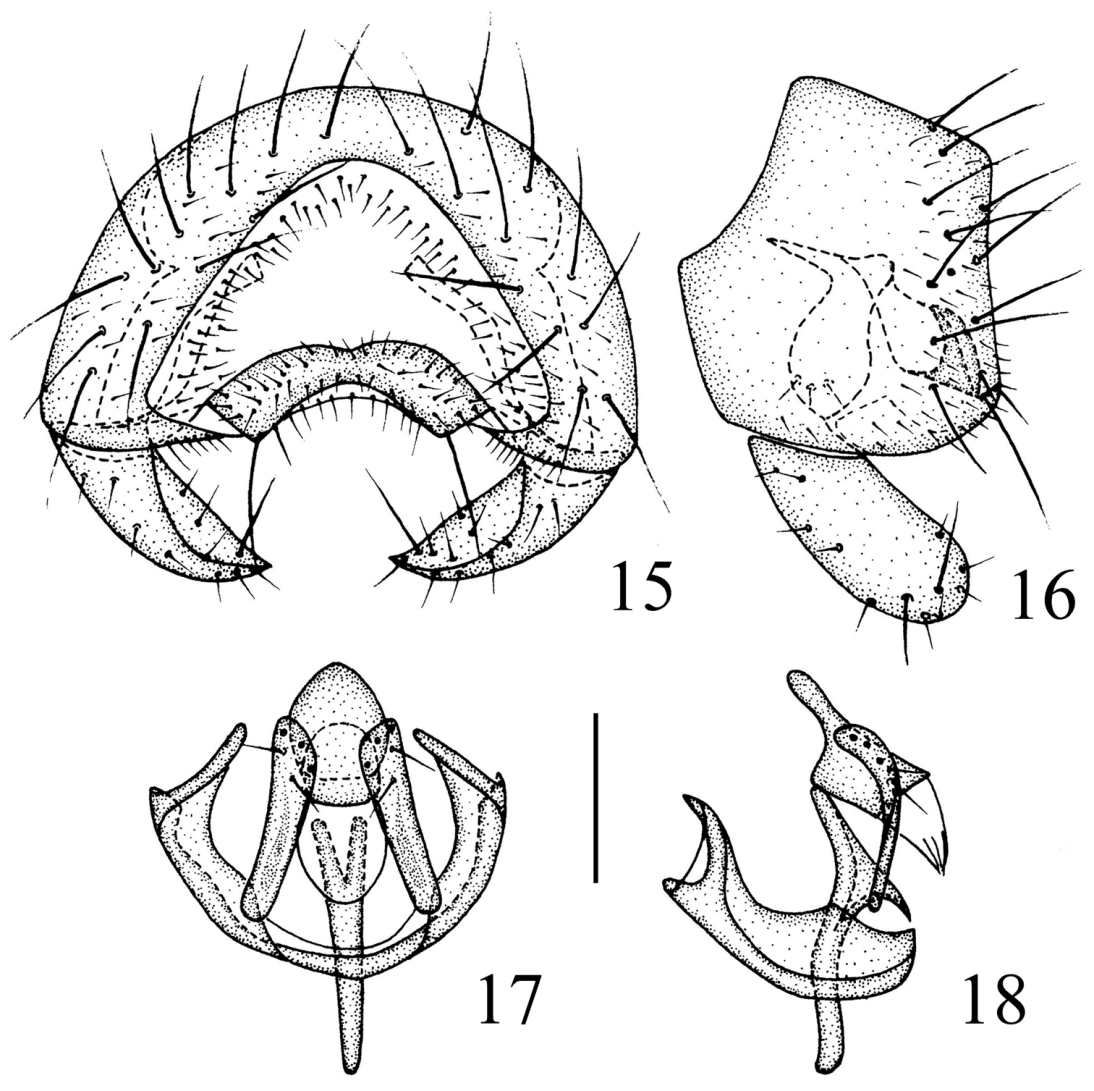
*Siphunculina shangyongensis* sp. n., holotype. **15** epandrium, posterior view **16** epandrium, lateral view **17** hypandrium and phallic complex, ventral view **18** hypandrium and phallic complex, lateral view. Scale bar: 0.1 mm.

*Male genitalia* (Figs 15–18): Epandrium blackish brown with long brown setulae; surstylus 0.6 times as long as epandrium in lateral view. Cercus short and broad, with a concavity on ventral margin. Gonite nearly rectangular, slightly narrowed basally; basiphallus slightly longer than wide, cylindrical; distiphallus short, membranous; phallapodeme long, distinctly projecting beyond hypandrium, with basal stalk narrow in lateral view. Hypandrium narrow, without two basal rounded projections, arms free and long, apex with internal projection long, external projection short.

***Female*.** Unknown.

##### Type material.

Holotype, ♂, China: Yunnan: Xishuangbanna, Shangyong, 7. V. 2007, leg. Wenliang Li (photographed and genitalia prepared). Paratype, 3 ♂♂, same locality and date as holotype, leg. Hui Dong (in 75% alcohol, deposited in CAU).

##### Distribution.

China (Yunnan).

##### Remarks.

The new species is somewhat similar to *S. bella* Kanmiya, but can be separated from the latter by the following features: hind tibia yellow except for middle 1/3 brown, tarsi yellow except for hind tarsomeres 2-4 brown, surstylus 0.6 times as long as epandrium in lateral view; in *S. bella*, the hind tibia is yellow with largely infuscate maculae medially, the tarsi are entirely yellow, the surstylus is as long as epandrium in lateral view (Kanmiya 1989).

##### Etymology.

The species is named after the type locality Shangyong.

#### Siphunculina funicola

Taxon classificationAnimaliaDipteraChloropidae

(de Meijere, 1905)

Figs 19–20 , 21–24

Siphonella funicola de Meijere, 1905: 160. Type locatity: Indonesia (Java).
Microneurum funicolum Becker, 1911: 141.
Liomicroneurum funicolum Duda, 1934: 112.
Siphunculina funicola (de Meijere): Becker et de Meijere, 1913: 303; de Meijere, 1918: 340; Sabrosky, 1977: 300; Cherian, 1977: 364; Kanmiya, 1989: 68.

##### Diagnosis.

Frons black with gray microtomentum. Ocellar triangle entirely shiny black with a broad median groove, reaching anterior margin of frons, with slightly pointed apex. Gena broad, 0.5 times as wide as first flagellomere. Antenna yellow except for dorsal margin of first flagellomere brown; arista with short pubescence. Thorax black with gray microtomentum. Scutellum with 4 pairs of scutellar setae on small tubercles. Cephalic and thoracic setae and setulae black; notopleurals 1+2. Legs black except for fore tibia, both ends of mid and hind tibiae and all tarsi yellow. Male genitalia (Figs 21–24): Surstylus shorter than epandrium in lateral view. Cercus 2 times as long as wide, deeply incised medially. Gonite long finger-like, basal 1/4 distinctly incised.

**Figures 19–20.**
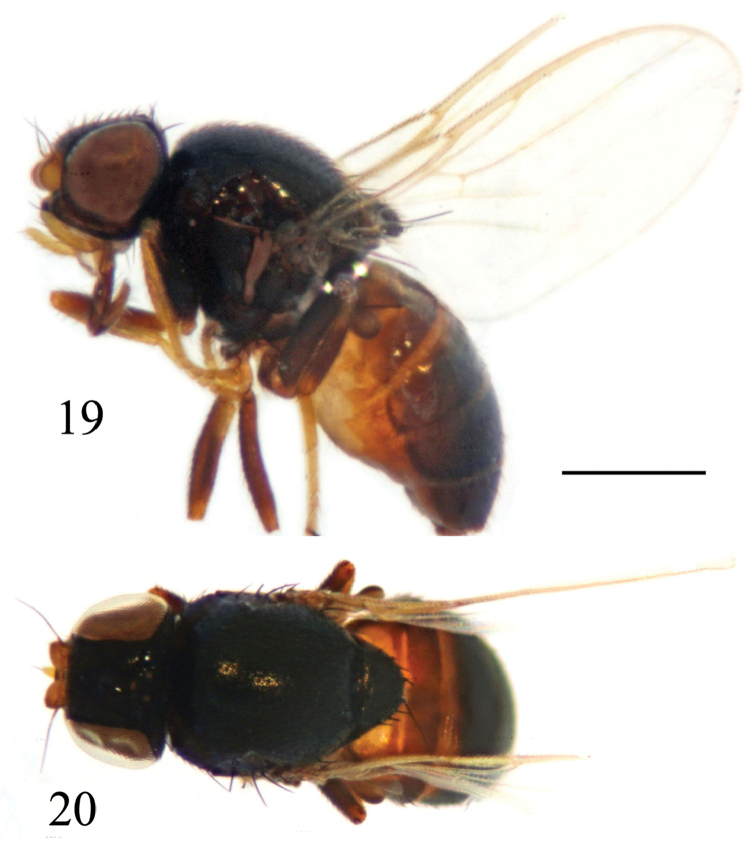
*Siphunculina funicola* (de Meijere), male. habitus **19** lateral view **20** dorsal view. Scale bar 0.05 mm.

**Figures 21–24.**
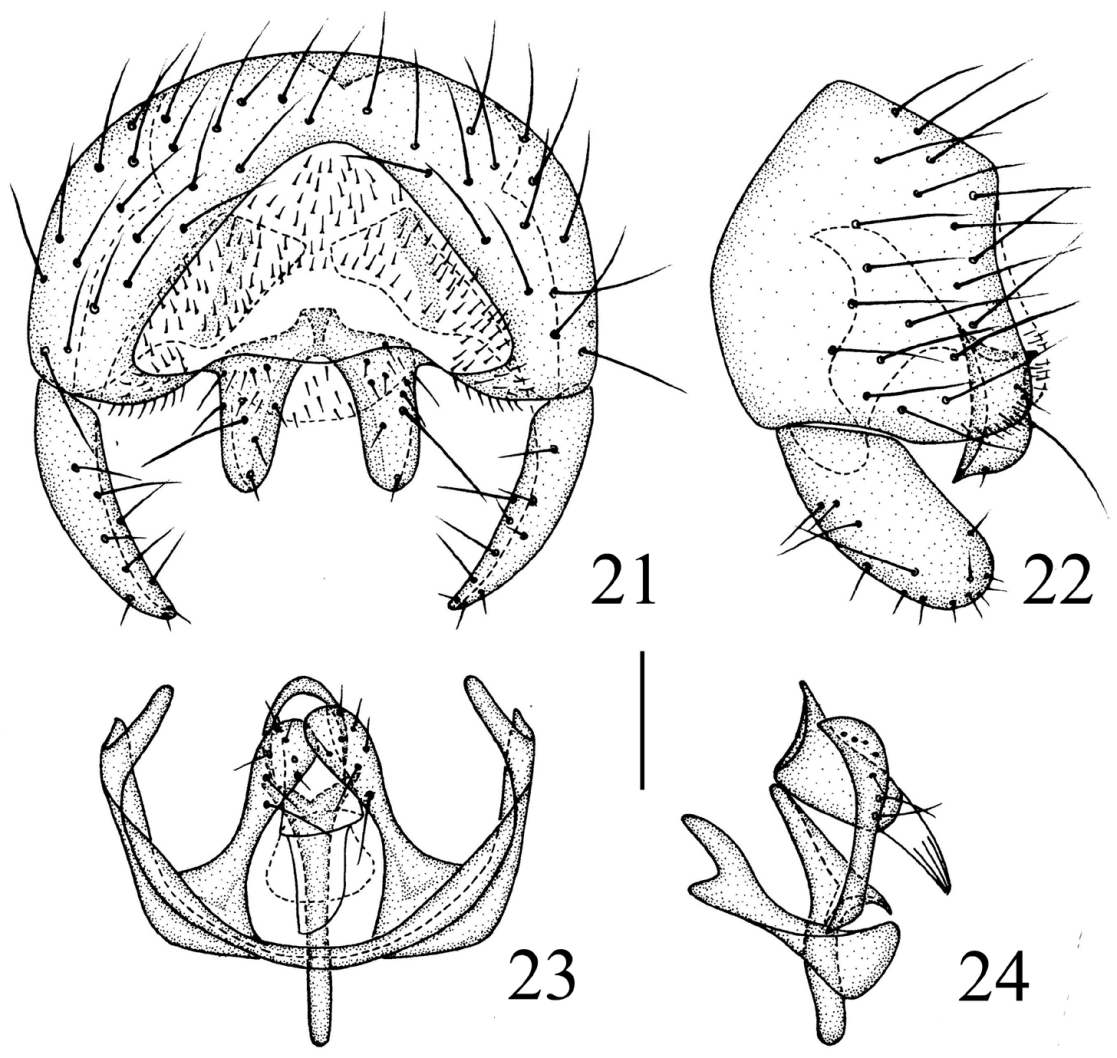
*Siphunculina funicola* (de Meijere), male. **21** epandrium, posterior view **22** epandrium, lateral view **23** hypandrium and phallic complex, ventral view **24** hypandrium and phallic complex, lateral view. Scale bar: 0.1 mm.

##### Specimens examined.

2 ♂♂, China: Hainan: Baisha, Hongmao, 19. V. 2007, leg. Ding Yang, 1 ♂, Hainan: Baisha, 22. V. 2007, leg. Kuiyan Zhang, 1 ♂, Hainan: Baisha, Yacha orchard, 19. IV. 2009, leg. Shan Huo (photographed and genitalia prepared).

##### Distribution.

China (Hainan); Cambodia, India, Indonesia, Malaysia, Sri Lanka, Vietnam, Thailand, Nepal.

##### Remarks.

This species has been called the Oriental eye-fly, predominantly inhabiting in the East and South Asian countries. The flies mass around men and cattle and cause considerable annoyance, and are responsible for spreading eye diseases. It is somewhat similar to *S. ceylonica* Kanmiya, but can be separated from the latter by the following features: ocellar triangle reaching anterior margin of frons, notopleurals 1+2, apical scutellar seta as long as scutellum, cercus twice as long as wide, deeply incised medially; in *S. ceylonica*, ocellar triangle reaching anterior 4/5 of frons, notopleurals 1+1, apical scutellar seta much shorter than scutellum, cercus short, widely incised medially (Kanmiya 1989).

## Supplementary Material

XML Treatment for Siphunculina

XML Treatment for Siphunculina bulbifera

XML Treatment for Siphunculina scalpriformis

XML Treatment for Siphunculina shangyongensis

XML Treatment for Siphunculina funicola
